# Phylogenetic Characterization and Genome Sequence Analysis of *Burkholderia glumae* Strains Isolated in Thailand as the Causal Agent of Rice Bacterial Panicle Blight

**DOI:** 10.3390/pathogens11060676

**Published:** 2022-06-11

**Authors:** Nootjarin Jungkhun, Antonio Roberto Gomes de Farias, Jutatape Watcharachaiyakup, Nuttima Kositcharoenkul, Jong Hyun Ham, Sujin Patarapuwadol

**Affiliations:** 1 Department of Plant Pathology, Faculty of Agriculture at Kamphaeng Saen, Kasetsart University Kamphaeng Saen Campus, Nakhon Pathom 73140, Thailand; nootjarin.j@rice.mail.go.th; 2 Rice Department, Chiang Rai Rice Research Center, Phan, Chiang Rai 57120, Thailand; 3 Center of Excellence in Fungal Research, Mae Fah Luang University, Chiang Rai 57100, Thailand; antonio.gom@mfu.ac.th; 4 Center for Agricultural Biotechnology, Kasetsart University Kamphaeng Saen Campus, Nakhon Pathom 73140, Thailand; jutatape.w@ku.th; 5 Center of Excellence on Agricultural Biotechnology: (AG-BIO/MHESI), Bangkok 10900, Thailand; 6 Department of Agriculture, Plant Pathology Research Group, Plant Protection Research and Development Office, Bangkok 10900, Thailand; nuttima.k@doa.in.th; 7 Department of Plant Pathology and Crop Physiology, Louisiana State University Agricultural Center, Baton Rouge, LA 70803, USA; jham@agcenter.lsu.edu

**Keywords:** bacterial panicle blight disease, *Burkholderia glumae*, multilocus sequence analysis, pan-genome, phage-integrated sequences

## Abstract

*Burkholderia glumae* is one of the most critical rice-pathogenic bacteria, and it causes bacterial panicle blight (BPB) in rice plants. In 2017, BPB symptoms were observed from rice fields in Chiang Rai, Northern Thailand. Sixty-one isolates obtained from the symptomatic panicles of rice were initially identified as *B. glumae* by polymerase chain reaction (PCR) using species-specific primers. Among them, six selected strains isolated from the susceptible *japonica* rice cultivar DOA2 were characterized in terms of morpho-physiology, pathology, phylogenetics, and genomics. Our genome sequence analysis of the six selected strains revealed the presence of multiple prophages, which may reflect the high level of diversity in this bacterial species through dynamic horizontal gene transfer processes, including phage infection. This notion was supported by the results of phylogenetic and phylogenomic analyses, which showed the formation of several subgroups not related to the years of isolation or the geographical origins. This study reports the isolation of *B. glumae* as the causal pathogen of BPB disease in *japonica* rice in Thailand and provides genomic resources to better understand the biology and diversity of this plant pathogenic bacterium. Further studies with a vast collection of *B. glumae* strains from various rice-growing regions around the world are needed to elucidate the evolution, variability, and lifestyle of the pathogen.

## 1. Introduction

*Burkholderia glumae*, causing bacterial panicle blight (BPB), is one of the most important rice-pathogenic bacteria because it affects the quality of the grain and spreads as a seed-borne pathogen to different regions [1,2]. Since it was first reported in Japan [3], this bacterium has become a global rice pathogen, as reported in major rice-growing areas. BPB (a.k.a. bacterial grain rot) has become a barrier to rice production due to the diversity of the pathogen, as well as the lack of effective methods to control the disease and resistant varieties [4,5]. This pathogen is more likely to spread in tropical and subtropical countries, and it is expected to be a more severe problem in the future if climate change is worsened [6].

The main symptom of *B. glumae* infection appears in the panicles as panicle blight and includes long gray lesions with edges of the scar showing red to dark brown colors. Other symptoms include light brown discoloration on the top part of the grains and dark red-brown discoloration on the lower part, which are accompanied by weight reduction and seed abortion, inducing yield loss that can reach up to 75% [1]. However, the diagnosis of BPB based solely on the symptoms can be inaccurate and unreliable due to other pathogens or abiotic stresses causing similar symptoms [7]. The identification of its causal pathogen can be made using the semi-selective SPG medium [8], polymerase chain reaction (PCR) based on species-specific primers [9], and the multilocus sequence analysis (MLSA) of housekeeping genes [10]. Nevertheless, newly collected bacterial isolates must be classified based on the polyphasic taxonomic data using diverse genotypic, chemotaxonomic, and phenotypic methods [11]. In this regard, studies have identified *B. glumae* successfully using polyphasic approaches [12,13].

With the development and popularization of high throughput sequencing and bioinformatics technics, genomics approaches have been widely used and accepted for the identification and taxonomy of bacteria [14,15,16,17], including the *Burkholderia* species [18,19]. However, despite the importance of *B. glumae* as a rice pathogen, the genome sequence information of this bacterial species is still relatively limited. The first complete whole-genome sequence *B. glumae*, the strain BGR1 from South Korea, was sequenced in 2009 [20]. Besides information on species delineation, genome information can also provide important insights into the adaptations and pathogenesis mechanisms of *Burkholderia* sp. in the environment and their plant hosts [21]. Genomic data have provided insights into the evolution of the *Burkholderia* species [22,23,24,25]. However, in the case of *B. glumae*, only 17 *B. glumae* strain genomes have been sequenced, annotated, and made available in the NCBI database (https://www.ncbi.nlm.nih.gov/genome/browse#!/prokaryotes/Burkholderia%20glumae, accessed on 22 February 2022), most of them from the USA and China.

In Thailand, *B. glumae* causing BPB was reported for the first time by Pet-amphai et al. [10]. This bacterium can be a severe problem in Thailand, and effective detection, identification, and management measures are imperative. Using morpho-physiological and phylogenomic analysis, this study identified *B. glumae* as the causal agent of BPB in japonica rice in Thailand and provided insights into the pathogen diversity and evolution.

## 2. Results

### 2.1. Isolation and PCR-Based Identification of Burkholderia glumae 

**Figure 1:**
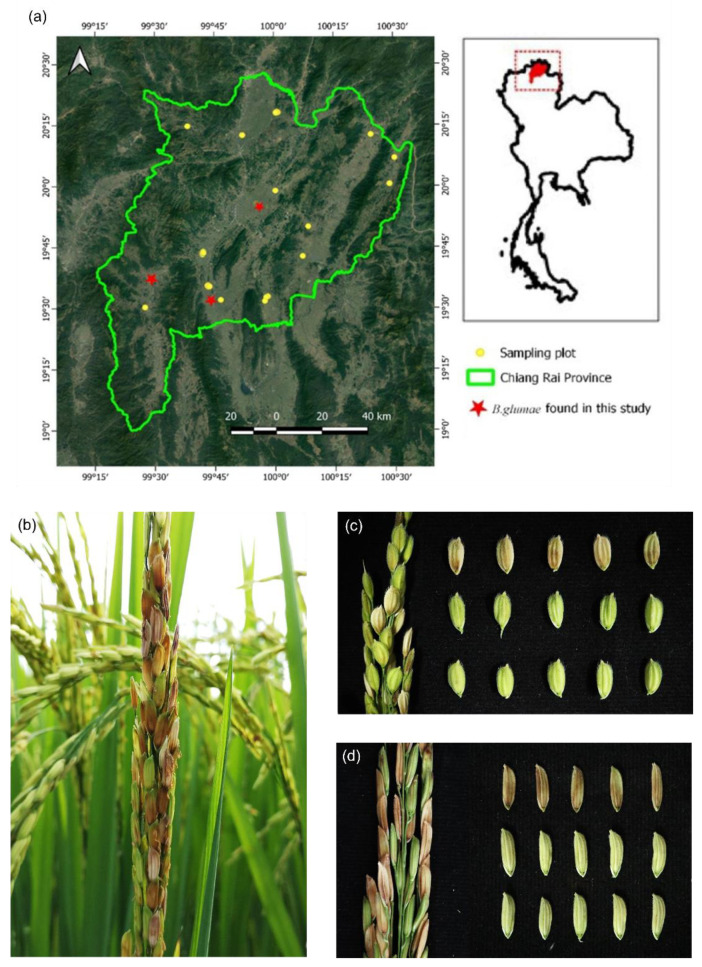
Graphical representation of the study area and typical symptoms of BPB disease in rice panicles and seeds found in infected fields in Chiang Rai districts. (**a**) The map of Chiang Rai province shows the sampling plots from which panicles showing blight symptoms were sampled (yellow points) and confirmed (red stars); (**b**) Panicle blight symptoms in the field; (**c**) Seed symptoms of *japonica* rice; (**d**) Seed symptoms of *indica* rice.

In the 2017 rice-growing season, we surveyed 289 fields in Chiang Rai province, Thailand, covering 18 districts (Figure 1a). The collected panicles presented the typical symptoms of *B. glumae* (dark red-brown lesions at the base of the seeds, light brown discoloration on the top part of the seed, and unfilled grains at the dough stage) (Figure 1a,b), which are easy to distinguish from healthy panicles in the field. Sixty-one *B. glumae*-like strains were obtained and screened using the *B. glumae* species-specific primers GL-13f and GL-14r. A DNA fragment of 400 bp was amplified from 44 strains (Table 1) and revealed in the agarose gel (data not shown).

### 2.2. Pathogenic and Biochemical Characterization of Six Selected Burkholderia glumae Strains

**Figure 2:**
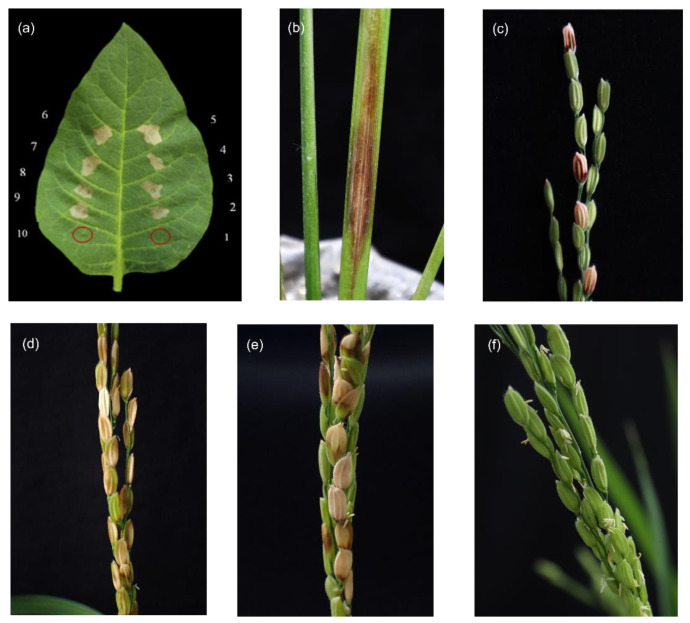
The symptoms shown after the inoculation of representative isolates on tobacco leaves and rice plants. (**a**) The hypersensitive response on tobacco leaves 24 h after inoculation (1 and 10 denote the negative controls, 0.85%NaCl and *Bacillus* sp. (TU), respectively, with red circles indicating the point of inoculation; 2 and 9 represent the positive control (*Burkholderia glumae* 1BGRE5-1); and 3 to 8 represent the six selected strains—60BGCRMSO1-5, 60BGCRMSO3-5, 60BGCRMSO3-9, 60BGCRMSO3-11, 60BGCRWC8-5, and 60BGCRPA10-1, respectively); (**b**) Sheath rot symptom in the rice tiller with the strain 60BGCRPA10-1 (five DAI); (**c**) Panicle blight symptom after inoculation of the strain 60BGCRPA10-1 in the rice tiller (forty-five DAI); (**d**) The rice panicle blight symptom at four DAI of the 60BGCRPA10-1 rifampicin-resistant strain at the rice flowering stage; (**e**) The rice panicle blight symptom at four DAI of the positive control strain 1BGRE5-1 at the rice flowering stage; (**f**) Asymptomatic panicle at four DAI with the negative control (0.85%NaCl) at the rice flowering stage.

The six strains isolated from japonica rice varieties and positive for PCR were able to induce an HR on tobacco leaves and cause disease in rice plants. All of them, along with the positive control, showed a positive HR on tobacco leaves 24 h after inoculation (Figure 2a). When inoculated into rice plants at the tillering stage, dark brown lesions with water-soaked halos on the inoculated sheath were observed at five DAI (Figure 2b); blight light brown discoloration on the top part of the seed and dark red-brown discoloration on the lower part when it emerged were observed at 45 DAI (Figure 2c). When inoculated in the rice panicles, the six Rifr-induced strains and the positive control strain (1BGRE5-1) caused typical symptoms of BPB on the panicles (Figure 2d,e), while no symptom was observed in the negative control (0.85%NaCl) (Figure 2f). In all cases, Koch’s postulations were confirmed by the re-isolation of the bacterial strains from the inoculated plants.

**Figure 3:**
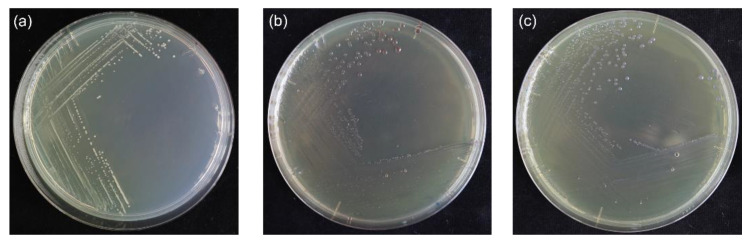
Colony morphology of the obtained strains. (**a**) Colony on NA media (strain 60BGCRMSO1-5); (**b**) S-PG medium, type A colony (strain 60BGCRMSO1-5); (**c**) S-PG medium, type B colony (strains 60BGCRMSO3-5, 60BGCRMSO3-9, 60BGCRMSO3-11, 60BGCRWC8-5, and 60BGCRPA10-1).

The six selected strains were Gram-negative, rod-shaped with a cell size of 0.49 to 0.53 × 1.62 to 2.06 μm, aerobic, and forming colonies of grayish-white or yellow with smooth margins within three days on NA media at 30 °C (Figure 3a). One strain (60BGCRMSO1-5) was typed as A colony on an S-PG medium presenting round colonies, smooth edges, and reddish-brown discoloration (Figure 3b), and the remaining (60BGCRMSO3-5, 60BGCRMSO3-9, 60BGCRMSO3-11, 60BGCRWC8-5, and 60BGCRPA10-1) were type B, with colonies with purple reflectors in the center of the magenta (Figure 3c). All the strains were positive for gelatin liquefaction and nitrate reduction and negative for starch hydrolysis, oxidase reaction, urease, and arginine dihydrolase (Supplementary Material Table S1). In addition, the strains were nonfluorescent under UV light, produced a yellow pigment in King’s B media, accumulated poly-β-hydroxybutyrate, grew under the conditions of 40 °C, NB media-supplemented with 3%, 5%, or 7% NaCl, and pH 8.0 and 9.0, but not pH 4.0 (Table S1).

### 2.3. Genome Features of Six Selected Burkholderia glumae Strains

The general whole-genome features of the six *B. glumae* genomes of the strains isolated from the japonica rice sequenced in this study are listed in Table 2. The sequenced reads were assembled into 191 (60BGCRMSO1-5), 162 (60BGCRMSO3-5), 173 (60BGCRMSO3-9), 174 (60BGCRMSO3-11), 183 (60BGCRPA10-1), and 186 (60BGCRWC8-5) contigs, which, after aliment with multi *B. glumae* references, built 27 (60BGCRMSO31-5), 12 (60BGCRMSO3-5), 10 (60BGCRMSO3-9), 11 (60BGCRMSO3-11), 13 (60BGCRPA10-1), and 14 (60BGCRWC8-5) scaffolds, with an average size of 6.5 Mb, CG content of 68.5%, and N50 of 131,659 bp (Table 2). The analysis of completeness using BUSCO indicated a high level of completeness (99.70–100%). Based on the results obtained from the prokka annotation, the respective genomes encode 5594, 5433, 5587, 5580, 5688, and 5752 proteins and 67, 62, 69, 69, 69, and 64 tRNA-coding genes.

The identification and comparison of the toxoflavin gene cluster of the Thai strains revealed that this cluster is conserved among the strains (Table S2). When comparing them with those strains from the database, a significant variation was observed in the *tofR*, *toxG*, and *toxI* genes. Indeed, the identity of BGR1 *tofR* vs. FDAARGOS_346, FDAARGOS_921, FDAARGOS_949, and LMG 2196 was 39.64%; BGR1 *toxG* vs. 411gr-6 55.2%; BGR1 *toxE* vs. 3252-8 71.51%; and BGR1 *ToxI* vs. 3252-8, Bp9029, and HN2 82.99%. The identity of the *tofI* and the other tox genes of the BGR1 with the other strains was always above 99% (Table S2).

### 2.4. Burkholderia glumae Strains from Thailand and Worldwide Harbor Specific Prophage Regions

**Figure 4:**
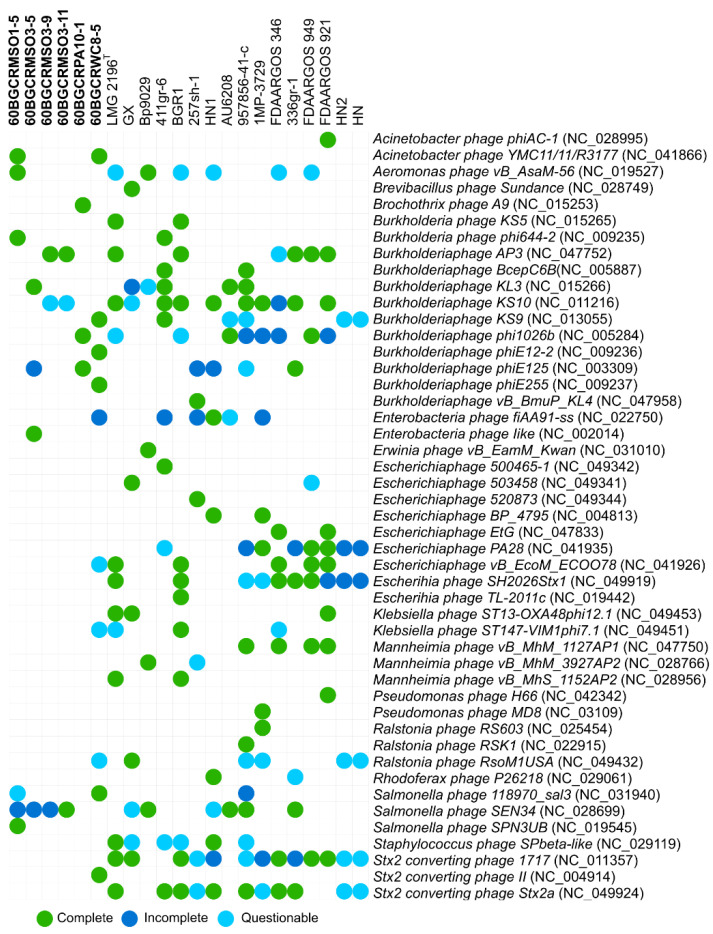
Phage-related sequences predicted in the assembled *Burkholderia glumae* genomes. Different colors indicate the completeness level of the prophage sequences based on the PHASTER scores. (A) green: “complete”; (B) blue: “incomplete”; (C) light blue: “questionable.” The strains sequenced in this study are labeled in bold.

The presence of phage-related sequences in the genome of *B. glumae* strains scanned using PHASTER revealed the presence of a different range of complete and incomplete prophage sequences (Figure 4). A total of 47 different prophages were found in the set of *B. glumae* genomes, from which 22 are present in the collection of Thai strains. Overall, the most prevalent phage species in the genomes were *Stx2-converting phage 1717*, *B. phage KS10*, *Salmonella phage SEN34*, *Stx2-converting phage Stx2a*, and *Escherichia phage SH2026Stx1*. The *S. phage SEN34* had the highest frequency in the Thai strains, with the bacterial strains 60BGCRWC8-5 and 60BGCRMSO1-5 harboring the highest number of prophage sequences (Figure 4). Interestingly, considerable differences in the occurrence and number of prophage sequences in the *B. glumae* genomes were observed, of which none were common to all bacterial strains.

### 2.5. Phylogenetic and Phylogenomic Analysis of Six Selected Burkholderia glumae Strains

**Figure 5:**
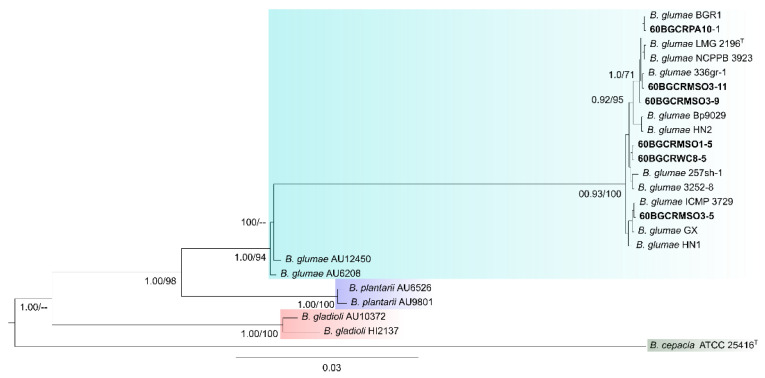
Combined Bayesian and Maximum Likelihood phylogenetic tree from the concatenated *atpD*, *gltB*, *gyrB*, *lepA*, *phaC*, *recA*, and *trpB* gene sequences of the six selected strains (60BGCRMSO1-5, 60BGCRMSO3-5, 60BGCRMSO3-9, 60BGCRMSO3-11, 60BGCRWC8-5, and 60BGCRPA10-1) isolated from japonica rice panicles presenting blight symptoms in Chiang Rai, Thailand, and the reference strains from the NCBI database. Values close to the nodes indicate Bayesian posterior probabilities and bootstrap support values for Maximum Likelihood and Bayesian analyses. Only values greater than 0.95 and greater than 70% are shown for the respective analysis. The scale bar represents the number of substitutions per site. The strains from this study are in bold. The *B. glumae* strains are highlighted in blue, *B. plantarii* in purple, *B. gladioli* in salmon, and *B. cepacia* (outgroup) in gray-blue.

The multilocus maximum likelihood (ML) and Bayesian phylogenetic inference of the concatenated sequences of the seven housekeeping genes (2770 nucleotides) exhibited similar structures (Figure 5). They revealed the genotypic relationships among the strains of *B. glumae* and the other rice-pathogenic species of *Burkholderia*, with all the strains isolated from japonica rice in Thailand grouping in the *B. glumae* clade. The strains 60BGCRMSO3-9 and 60BGCRMSO3-11 (Mae Suai District) and 60BGCRPA10-1 (Phan District) clustered with the strains 336gr-1 (USA), BGR1 (South Korea), NCPPB 3923 (Vietnam), ICMP 3923 (Vietnam), LMG 2196T (Japan), Bp9029 (Puerto Rico), and HN2 (China). The strains 60BGCRMSO1-5 (Mae Suai District) and 60BGCRWC8-5 (Wiang Chai District) grouped with the strains 257sh-1 (USA) and 3252-8 (Colombia), and the strain 60BGCRMSO3-5 (Mae Suai District) formed a distinct group with the strains GX, HN1 (China), 3252-8 (Colombia), and ICMP 3729 (Japan) (Figure 5). The strain from the species *B. gladioli* and *B. plantarii* formed clades corresponding to each species, and the *B. cepacia* ATCC 25416T rooted the tree, as expected (Figure 5).

**Figure 6:**
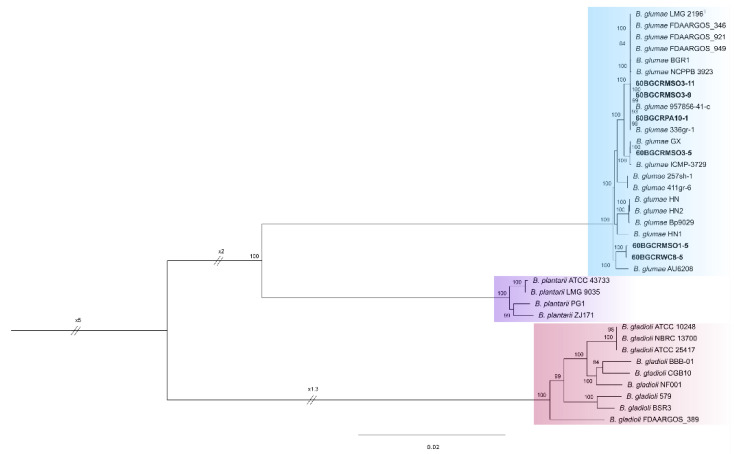
Maximum likelihood pan-genome phylogeny of the rice-pathogenic *Burkholderia* genomes based on 529 core genes. Values close to the nodes indicate the bootstrap support values. Only values greater than 0.95 and greater than 70% are shown for the respective analysis. The scale bar represents the number of substitutions per site. The strains from this study are in bold. The *B. glumae* strains are highlighted in blue, *B. plantarii* in purple, and *B. gladioli* in salmon.

The assessment of the pan genome using the Roary pipeline identified a total of 529 single-copy orthologous genes (core genome) shared by the six genomes from Thailand, along with 30 genomes from the NCBI database, including *B. glumae* (17), *B. gladioli* (9), and *B. plantarii* (4) (Table S3). The core genes were used to build an unbiased maximum likelihood phylogeny. The phylogenetic analysis of the concatenated 529 core genes alignment revealed that the *B. glumae* strains formed a well-resolved clade, including the six Thai strains (Figure 6), with *B. gladioli* and B. *plantarii* forming the other single clades in the phylogenetic tree. Within the *B. glumae* clade, the Thai strains also clustered into specific groups: the 60BGCRMSO3-9, 60BGCRMSO3-11, and 60BGCRPA10-1 grouped with the type strain LMG 2196T and the strains FDAARGOS_346, FDAARGOS_921, FDAARGOS_949, BGR1, NCPPB 3923, and 336gr-1; the strains 60BGCRMSO3-5 formed a group with GX and ICMP-3729; the strains 60BGCRMSO1-5 and 60BGCRWC8-5 clustered with the strain AU6208. The other *B. glumae* strains included in the analysis formed two other groups, one comprising 257sh-1 and 411gr-6 and the other comprising the HN, HN1, HN2, and Bp9029 strains. Subclades were also formed by the *B. gladioli* and *B. plantarii* strains (Figure 6); however, for the three species, the grouping is not related to the host, environment, or lifestyle.

### 2.6. Taxogenomic analysis of Six Selected Burkholderia glumae Strains

The pairwise ANI and *is*DDH calculated for the 36 rice pathogenic bacteria showed values consistent with their phylogenetic relationships obtained by the MLSA (Table S4). The strains grouped in the same phylogenetic group had high ANI and *is*DDH values, consistent with the 95% and 70% thresholds for the species delineation based on the ANI and *is*DDH, respectively (Table S4). Among the *B. glumae* strains, the values of ANI ranged from 99.43% to 100%, while the *is*DDH fluctuated from 95.1% to 100%. Between *B. glumae* and the other species, the ANIm values fluctuated from 92.65% to 93.19% (*B. glumae* vs. *B. plantarii*), 87.92% to 88.40% (*B. glumae* vs. *B. gladioli*), 29.3% to 31.7% (*B. glumae* vs. *B. gladioli*) and 43.94% to 47.4% (*B. glumae* vs. *B. plantarii*). The ANIm and *is*DDH values between the strains of the other rice pathogenic *Burkholderia* were also consistently above 98% and 92.4% (between *B. plantarii* strains) and 99.04% and 79.9% (between *B. gladioli* strains), respectively (Table S4).

### 2.7. Core Genome Characterization of Six Selected Burkholderia glumae Strains

**Figure 7:**
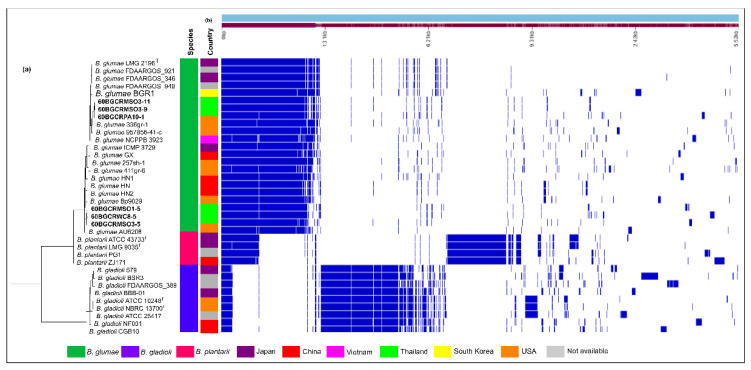
Visualization of the pan-genome analysis of the 36 rice pathogenic *Burkholderia* performed by Roary. (**a**) Maximum likelihood tree based on core and accessory presence/absence genes in the genomes; (**b**) Pan-genome distribution of the pan-genome genes based on the core and accessory genes, showing the phylogenetic relatedness of the strains by blue (present) and white (absent).

The pangenome analysis indicates a low level of conservation among the rice pathogenic *Burkholderia*, with the core genome (genes present in 99% or more strains) comprising only 529 genes. However, considerable gene conservation exists within each species (Figure 7), indicating the different adaptability of the pathogens. Based on the KEGG functional annotation of the core genes of the genomes sequenced in this study, 35.81% (60BGCRMSO3-5) to 58.14% (60BGCRWC8-5) of the core genomes do not match the KEGG orthologs genes, indicating a different evolutionary history of the strains—except for the strains 60BGCRMSO3-9 and 60BGCRMSO3-1, which had around 58% of the core genomes, with a corresponding ortholog in the KEGG database (Table S5). As shown in Figure 7, even though there is a considerable level of conservation among the *B. glumae* genomes, significant variation is observed, forming defined groups based on the presence and absence of genes (Figure 7a,b). Similar arrangements are observed for the *B. plantarii* and *B. gladioli* genomes. It was also observed that each strain contains a set of specific genes, such as those encoding for prophage sequences, which are strain-specific.

## 3. Discussion

The bacterium *B. glumae*, causing the bacterial panicle blight (BPB) disease, is a seed-borne pathogen regulated under the Thai Plant Quarantine. In Thailand, *B. glumae*-like- strains were first isolated by Pet-amphai et al. [10]. Therefore, the survey, detection, and accurate identification of this pathogen in the main rice-producing areas should be carried out to choose proper control strategies [6]. In this context, this study surveyed the panicle blight symptoms in the 18 districts of Chiang Rai province (Figure 1a), which stands out as the biggest rice producer in the upper-North part of Thailand. Sixty-one *Burkholderia*-like bacterial strains were isolated from samples presenting blight symptoms—dark red-brown lesions at the base, light brown discoloration on the top part of the seed, and unfilled grains [2,3,12] (Table 1, Figure 1b–d).

Because of the high similarity of *B. glumae* cultures with other *Burkholderia* spp., the correct identification of this bacterium is a challenge [2], and a polyphasic approach should be used for its correct documentation [11]. In our study, the bacterial strains isolated from the collected japonica rice samples were characterized using phenotypic, biochemical, molecular, and genomic methods. The grayish-white or yellow color of the isolated colonies (Figure 3a) was consistent with previous studies reporting *B. glumae* and the production of the bright yellow toxoflavin, a virulence factor [6,26,27,28,29]. The colonies were also typed as type A or type B (Figure 3b,c) on the differential S-PG medium, which has been reported in studies of the morphological characterization of *B. glumae* colonies [8,10,13]. The genetic basis of these phenotypes is unknown; thus, genetic studies may provide information on whether the type A and B phenotypes give any advantages to the adaptation and/or virulence of *B. glumae* to rice.

Regarding identification, several molecular methods based on PCR have been developed and established to detect *B. glumae* [4,9]. Forty-four strains were positive for *B. glumae*, with the species-specific PCR primers GL-13f and GL-14r designed for the 16S-23S rDNA ITS, which amplified 400 bp DNA [9,10,29]. In addition, six selected strains isolated from japonica rice had an HR reaction on the tobacco leaves, and Koch’s postulations proved the association of the strains (Figure 2), as previously reported [10,30]. The physiological and biochemical characteristics of the strains (Table S1) also agree with other *B. glumae* studies [10,13,31], except for pH, as Pet-amphai et al. [10] reported the growth of Thai strains at pH 9.0, probably because of some degree of genotypic variations or environmental adaptations.

Once detected and characterized using the above approaches, six strains (60BGCRMSO1-5, 60BGCRMSO3-5, 60BGCRMSO3-9, 60BGCRMSO3-11, 60BGCRPA10-1, and 60BGCRWC8-5) were selected and subjected to whole-genome sequencing, characterization, and phylogenetic analysis. The genome characteristics of the sequenced strains were consistent with the previously sequenced strains of this pathogen (e.g., [20,21,32,33]), with a high level of completeness (Table 2). The toxoflavin gene cluster of the six genomes was identified and compared with the other publicly available *B. glumae* genomes. As expected, this gene cluster is present in all the strains; however, a significant variation between the *tofR*, *toxG*, and *toxI* genes among some strains was observed (Table S2), which may reflect the different levels of virulence of these strains. In this regard, when compared with the other *B. glumae* strains, the high rice virulent strain 411gr-6 [5,32] has a significant difference in the aminoacid sequence of the *toxG* (55.2%), while the other strains showed ≥99.6% of identity (Table S2). Similarly, a significant difference was observed in the aminoacid sequence of the LysR-type regulator *toxR* of the FDAARGOS_346, FDAARGOS_921, FDAARGOS_949, and LMG 2196 strains. This gene regulates the expression of both the *toxABCDE* operon and *toxFGHI* operon, as well as *toxJ* expression, which are regulated by quorum-sensing [26]. Thus, it may also reflect in the virulence of these strains. The variation in the aminoacid sequence identity was also observed in the *toxI* sequence of the 3252-8, Bp9029, and HN2 strains. Nevertheless, this gene seemed not to affect phytotoxin production and transport [26]. Interestingly, the nonpathogenic strain 257sh-1 and the low pathogenic 957856-41-c showed similar identities to the *tof*/*tox* genes of the other strains, indicating that the mechanisms underlining the genetic basis of *B. glumae* still require many studies.

A high and a variable number of prophage sequences were predicted in the six Thai genomes and the other sequenced genomes used for comparisons (Figure 4). In this regard, Varani et al. [34] showed that prophages can play a significant role in the evolution of the bacterial genome. They can cause rearrangements and can lead to alterations in the expression of phage-encoded proteins, resulting in the establishment of irreversible lysogeny [35] and the diversification of the bacterial genome architecture, and in many strains, they represent a significant fraction of the strain-specific DNA sequences [34,36]. Remarkably, phages have also been reported as efficient biocontrol agents against *B. glumae* [37,38]. Thus, investigations on the contribution of these agents in the lifestyle, such as pathogenic and nonpathogenic strains, and the diversity of *B. glumae* are the subjects of future work.

Seven housekeeping gene sequences (*atpD*, *gltB*, *gyrB*, *lepA*, *phaC*, *recA*, and *trpB*) [39] were extracted from whole-genome sequence annotations and subjected to Bayesian and ML MLSA inference with other *B. glumae* strains and the rice-pathogenic *B. gladioli* and *B. plantarii* strains, using their sequence data in the GenBank database. Bayesian and ML phylogenetic analyses grouped the six strains with the reference *B. glumae* strains (Figure 5). Interestingly, the strains 60BGCRMSO3-5, 60BGCRMSO3-9, and 60BGCRMSO3-1, collected in the same field, were grouped in different groups, indicating a genetic variability among the strains independent of the geographic locations (Figure 5). Otherwise, as shown in Figure 4, a large number of prophages with significant variability were observed in the genomes of the Thai strains. This is more evident in Figure 7, which shows the presence/absence matrix of 529 single-copy orthologous genes and strains from the same location clustered in different groups. Genomic regions related to prophage sequences are strain- or species-specific. Knowing that prophage infection can lead the evolution, create a more genetically diverse bacterial population, and increase their capacity to adapt to new niches and different hosts and environments [34,36], the variability observed in the Thai *B. glumae* strains may be related to the prophage insertions in the genomes.

The genetic variability observed among the Thai *B. glumae* is consistent with previous studies conducted with strains from the USA and South Korea. In the USA, genetic variations among *B. glumae* strains were detected by Karki et al. [5]. Using DNA fingerprinting analyses, the authors observed that the strains were clustered in different phyletic groups. Based on the transposase-based PCR (Tnp-PCR) genomic fingerprinting, 138 *B. glumae* Korean strains were grouped into 11 clusters and 3 divisions [40], where strains from the same geographical location were clustered into different groups. Some isolates were widely distributed in the country, which can be the result of the settlement and adaptation of those strains in the rice fields. In Thailand, this pathogen is relatively new [10], and rice is planted two times a year in the same field. Therefore, data on the varieties, the cultivation history of the fields [40], cultural practices, and the sources of seeds are required to understand the diversity better and control this bacterium.

On the other hand, Pet-amphai et al. [10] used MLSA and showed that ten rice panicle-associated bacteria from Thailand formed a single clade. Our study showed that the six strains formed a single clade based on both Bayesian and ML analyses, with three subgroups with strains from other countries (Figure 5), indicating a degree of diversity. Interestingly, while the strains isolated from rice formed a homogeneous group, two human clinical strains isolated from the lung (AU6208) [41] and AU12450 (https://pubmlst.org/bigsdb?page=info&db=pubmlst_bcc_isolates&id=913, accessed on 03 March 2022) formed a basal subclade, indicating an unmistakable evolution and adaptation of the pathogen. Notably, Cui et al. [42] reported that the clinical strain AU6208 presents a high level of virulence in rice plants.

Genomic and pangenome analyses have become important tools for identifying and characterizing microbial organisms. We used phylo and taxogenomic approaches for their correct taxonomic placement and gave clues as to their adaptation and evolution. The phylogeny of the 529 single-copy orthologous showed that the *B. glumae* strains form divergent groups (Figure 6), reflecting the KEGG orthologs annotations (Table S5) and confirming the assumption that their core genes have different evolutionary origins. In addition, the ANIm values were consistent with the phylogenetic and phylogenomic studies (Table S4), with a threshold of 95 for the species delineation based on ANI [17], confirming the identity of the strains.

## 4. Materials and Methods

### 4.1. Sample Collection, Isolation, and PCR-Based Identification

Symptomatic rice panicles were collected during the survey of 289 fields in 2017 in Chiang Rai province, Thailand. From each panicle, five symptomatic seeds were subsampled for the bacteria isolation. For that, the seeds were washed in tap water for 5 min, soaked in 70% ethanol for 5 s, rinsed with sterilized water two times, and dried on the sterilized tissue paper. Then, the seeds were cut into short pieces with sterilized scissors, put in a 1.5 mL Eppendorf tube with 1.0 mL of sterilized water, vortexed for 10 sec, and kept at room temperature for 15 min. The obtained suspension was then cross streaked on Nutrient agar (NA) plates and incubated at 30 °C for 48 h. Each *B. glumae*-like colony selected was stored in 1.5 mL of 20% glycerol (at −20 °C for routine use and −80°C for long-term storage). The identity of the isolates was confirmed by the amplification of a 400 bp fragment of the bacterial 16S-23S rDNA internal transcribed spacer (ITS), using the pair of primers GL-13f (ACACGGAACACCTGGGTA) and GL-14r (TCGCTCTCCCGAAGAG) for *B. glumae* [9], following the standard protocol. 

### 4.2. Hypersensitive Response and Pathogenicity Test

Six bacterial strains isolated from the japonica rice positive for PCR (60BGCRMSO1-5, 60BGCRMSO3-5, 60BGCRMSO3-9, 60BGCRMSO3-11, 60BGCRWC8-5, and 60BGCRPA10-1) were selected for pathogenicity tests and further characterizations. Initially, the bacterial strains were tested for their ability to induce a hypersensitive response (HR) on tobacco leaves (*Nicotiana tabacum* cv Xanthi), according to Furuya et al. [30], followed by a pathogenicity test on the rice plants. The pathogenicity of the isolates was tested on rice plants (cultivar DOA2), which were obtained according to Jungkhun et al. [38] (2021). The plants were inoculated at the tillering stage using a modified method from Pet-amphai et al. [10]. Briefly, the bacterial strains were cultured on NA at 30 °C for 48 h, followed by the adjustment of the bacterial suspension to the concentration of OD600 = 0.2 (3 × 10^8^ CFU/mL) with sterile 0.85% NaCl using a Spectrophotometer (Spectronic, Instruments Inc., Melville, NY, USA). The tiller of the rice plants was infiltrated with 0.25 mL of each bacterial suspension at the tillering stage using a 1 mL syringe. The disease was evaluated seven days after inoculation (DAI).

For the inoculation of the rice plants at the flowering stage, the bacterial strains were induced to rifampicin resistance by a successive culture in NA medium plates supplemented with a gradual concentration of rifampicin (Rif) (50, 100, 150, 200, and 250/µg.mL^−1^), according to Glandorf et al. [43]. Following this, the rice panicles were sprayed with 3 mL of a suspension of the selected Rif-resistant bacteria at the concentration of 10^8^ CFU/mL, prepared as previously described, kept in a humid chamber for 24 h, and evaluated for the symptoms at four DAI. In both stages, the bacteria were re-isolated and tested for the amplification of the rRNA ITS region, as previously described, to verify Koch’s postulation.

### 4.3. Phenotypic and Biochemical Characterization

Phenotypic and biochemical characterizations were carried out with bacteria grown for 48 h at 30 °C on NA media. Gram staining; poly-β-hydroxybutyrate accumulation; the oxygen relationship; the presence of oxidase; the production of fluorescent pigment; nitrate reduction to nitrite; the urease test; the production of dihydrolase of arginine; the hydrolysis of starch; the hydrolysis of gelatin; growth at 40 °C; growth at pH 4, 8, and 9; growth at 3%, 5%, and 7% NaCl; growth on YDC media; and growth on S-PG media were performed, following the protocols described by Schaad et al. [7].

### 4.4. Whole-Genome Sequencing, Assembly, and Annotation

The strains were subjected to genomic DNA extraction using the PrestoTM Mini gDNA Bacteria Kit (Geneaid Biotech Ltd., New Taipei City, Taiwan), following the manufacturer’s protocol, and sent to the Vishuo Biomedical (Bangkok, Thailand) LTD for whole-genome sequencing. A paired-end library (2 × 150 bp) was constructed, and the genome sequencing was performed in an Illumina HiSeq® 2500 Sequencing System, HiSeq Control Software v2.2.68 (Illumina, San Diego, CA, USA). The quality of the reads was analyzed by FastQC [44], and the low-quality reads and adaptors were treated using Trimmomatic v.0.39 [45], keeping all of the good-quality paired reads (Phred quality score Q ≥ 30). De novo assembly was performed with the trimmed reads dataset using the SPAdes v. 1.10 [46] implemented in the Unicycler pipeline v.0.4.9 [47], which was used for the automatic improvement of the assembly. The assembled contigs larger than 500 bp were scaffolded in Medusa v.1.6 [48], using the *B.glumae* genomes with the “complete” status in the NCBI database as a reference (Table S3). The completeness of the genomes was accessed using BUSCO v.5.2.2 [49] and annotated using Prokka v1.14.5 [50]. The prediction of phages was performed using the PHASTER [51], and the functional annotation of the core genes (see below) was realized by comparing protein sequences against the KEGG database using BLASTKOALA [52]. Computational analyses of the toxoflavin gene cluster were performed by BLASTP searches, with 60% of coverage, using the sequences from the BGR1 strain obtained from the literature and downloaded from the UniProt database as a reference [53]. The gene sequences were then aligned with MAFFT v. 7.310 [54] and visualized in MEGA v. 11 [55].

### 4.5. Phylogenetic and Phylogenomic Analysis

Phylogenetic analyses were performed based on a multilocus sequence and phylogenomic analysis. The sequences of the housekeeping genes, viz., *atpD*, *gltB*, *gyrB*, *lepA*, *phaC*, *recA*, and *trpB* [56], from the annotated genomes and other rice pathogenic *Burkholderia* were retrieved from their genomes available in the NCBI database (Table S3) and subjected to a multilocus sequence analysis (MLSA). The downloaded gene sequences, along with those obtained from the whole genome sequence from this study, were individually aligned and trimmed using the online servers MAFFT (https://mafft.cbrc.jp/alignment/server/, accessed on 25 March 2022) [54] and Gblocks v. 0.91b [57], respectively. The trimmed sequences were subjected to the software jModelTest2 v. v2.1.10 [58] for the nucleotide substitution model calculations. The alignments were concatenated and subjected to Maximum Likelihood (ML) and Bayesian phylogenetic analyses. The ML was performed using RaxML v.8.2.12 [59], with 1000 bootstrap replicates. The Bayesian analyses were conducted using MrBayes v. 3.1.2 [60], employing four simultaneous Markov chain Monte Carlo (MCMC) simulations for 3,000,000 generations, with trees sampled at every 300th generation. The first 25% of the obtained trees, representing the burn-in phase, were discarded, and the remaining trees were used to calculate the posterior probabilities in the majority rule consensus tree. The phylogenetic trees were visualized with FigTree v1.4.4 software [61] and edited using the Inkscape open-source vector graphics editor v.1.1.1 (https://inkscape.org/, accessed on 27 March 2022). 

For the phylogenomic analysis, the *B. glumae* complete genomes were downloaded from the NCBI (Table S3), and their level of completeness was checked by BUSCO v.5.2.2 using the burkholderiales_odb10.2021-02-23 database as a reference (https://busco-data.ezlab.org/v5/data/lineages/, accessed on 14 February 2022). The genomes presenting more than 95% completeness were used for the subsequent analysis (Table S3). Roary v3.13.0 [62] was used to perform a pangenome analysis using the default configurations, except that the core genes were automatically aligned with MAFFT v. 7.310 [54] using the *-e --mafft* parameter. A maximum-likelihood (ML) phylogenomic tree from the core genome was built using IQ-TREE v.2.0.4 [63], using the automatic selection of the nucleotide substitution model on ModelFinder for the selection of the best fitting DNA substitution model [64]. The node and branch support were assessed with an ultrafast bootstrap [65,66] and an SH-like approximate likelihood ratio test (SH-aLRT) [67] using 100,000 replicates. The ML tree was visualized as previously described.

### 4.6. Taxogenomic Analysis

The Average Nucleotide Identity (ANI) was calculated using pyani v.0.11 Python3 [68] through the whole genome alignments with Mummer (ANIm) [17]. Following this, in silico DNA-DNA hybridization (*is*DDH) between the whole genomes was determined using the webserver Genome-to-Genome Distance Calculator (GGDC) v.3.0 (http://ggdc.dsmz.de/distcalc2.php, accessed on 16 March 2022) [14,15] under the local alignment BLAST+ tool, with the results computed by formula 2 (DDH estimates based on identities/HSP—High Scoring Pair length [14,15].

## 5. Conclusions

Our findings considerably contribute to increasing the knowledge about *B. glumae*. Six bacterial strains isolated from *japonica* rice panicles from Chiang Rai, Thailand, were confirmed as the causal agents and identified as *B. glumae* using morpho-molecular and genomics approaches. Morpho-physiological, molecular, and pathological approaches were employed to preliminarily identify this pathogen, and MLSA and genomic methods, viz., ANI and *is*DDH, were used for more reliable results for verification. Furthermore, the analysis of the genome sequences provided insights into the biology of *B. glumae*, which underlies its pathogenicity, virulence, diversity, adaptation, and evolution. However, further and more detailed genetic and evolutionary studies of *B. glumae* are needed to elucidate the mechanisms of this pathogen.

## Figures and Tables

**Table 1:** Summary of the rice bacterial panicle blight disease survey in Chiang Rai in 2017.

District	Surveyed Fields	Fields with Symptomatic Samples	Panicle Samples	Bacteria Isolates	Positive PCR Result
Mae Suai	30	12	123	12	7
Wiang Chai	15	4	40	13	11
Pa Daet	25	3	30	5	4
Mae Chan	18	2	20	9	9
Chiang Khong	16	16	160	4	2
Chiang Saen	17	4	31	1	1
Wiang Kaen	3	3	23	7	5
Phan	44	16	142	2	2
Thoeng	17	7	59	1	1
Mae Lao	6	6	38	3	2
Phaya Mengrai	14	2	20	1	0
Wiang Chiang Rung	14	5	37	1	0
Mae Fa Luang	4	4	23	2	0
Muang Chiang Rai	16	6	56	0	0
Mae Sai	33	3	22	0	0
Wiang Pa Pao	7	5	30	0	0
Kun Tan	7	4	41	0	0
Doi Luang	3	3	30	0	0
**Total**	**289**	**105**	**925**	**61**	**44**

**Table 2:** Genome features and statistics of the sequenced and assembled *Burkholderia glumae* genomes.

Feature	60BGCRMSO31-5	60BGCRMSO3-5	60BGCRMSO3-9	60BGCRMSO3-11	60BGCRPA10-1	60BGCRWC8-5	Average
No. contigs	191	162	173	174	183	186	178
Size (Mb)	6.45	6.30	6.56	6.56	6.59	6.59	6.51
GC content (%)	68.54	68.76	68.39	68.39	68.39	68.36	68.42
N50 (bp)	113,987	110,811	161,337	161,338	109,662	132,818	131,659
No. scaffolds	27	12	10	11	13	14	14.5
Size (Mb)	6.46	6.31	6.57	6.57	6.59	6.59	6.52
Completeness (%)	99.90	99.70	99.90	99.90	99.90	100.00	99.89
No. coding sequences	5594	5433	5587	5580	5688	5752	5606
No. tRNA	67	62	69	69	69	64	67

## Data Availability

All data supporting the conclusions of this article are included in this article and its additional files. The Whole Genome Shotgun project has been deposited at DDBJ/ENA/GenBank under the accessions JAMQEL000000000, JAMQEM000000000, JAMQEN000000000, JAMQEO000000000, JAMQEP000000000, and JAMQEQ000000000. The versions described in this paper are version JAMQEL000000000, JAMQEM000000000, JAMQEN000000000, JAMQEO000000000, JAMQEP000000000, and JAMQEQ000000000.

## Supplementary Materials

The following supporting information can be downloaded at: https://www.mdpi.com/article/10.3390/pathogens11060676/s1, Table S1: Table S1. The morphological and biochemical results of the six selected strains’ isolated panicles presenting blight symptoms from Chiang Rai; Table S2: Table S2. Blast identity (%) of the *Tof* and *Tox* genes of the *Burkholderia glumae* strains, using the BGR1 strain as a reference; Table S3: List of rice pathogenic *Burkholderia* accessions retrieved from the NCBI database used for multilocus sequence phylogeny and pangenome analysis; Table S4: Pairwise average nucleotide identity and in silico DNA-DNA hybridization between *Burkholderia* rice pathogenic bacteria; Table S5: KEEG annotation of the core genes of the six *Burkholderia glumae* strains sequenced for this study.
